# Transition Metal Catalysts for Atmospheric Heavy Metal Removal: A Review of Current Innovations and Advances

**DOI:** 10.3390/molecules28227620

**Published:** 2023-11-16

**Authors:** Qiang Ma, Xianglong Zhang, Jie Li, Yingjie Zhang, Qingyuan Wang, Li Zeng, Yige Yang, Yonghong Xie, Jin Huang

**Affiliations:** 1 Sichuan Provincial Engineering Research Center of City Solid Waste Energy and Building Materials Conversion & Utilization Technology, Key Laboratory of Drinking Water Source Protection in Chengdu Basin of Sichuan Province, Chengdu University, Chengdu 610106, China; maqiang@cdu.edu.cn (Q.M.); zhangxianglong@stu.cdu.edu.cn (X.Z.); huangjin_cd@cdu.edu.cn (J.H.); 2 College of Agriculture and Biological Science, Dali University, Dali 671000, China; zyj@dali.edu.cn; 3 Sichuan Academy of Eco-Environmental Sciences, Chengdu 610091, China; 4 Sichuan Province Environmental Monitoring Station, Chengdu 610091, China

**Keywords:** heavy metals, life cycle assessment, kinetic modeling

## Abstract

Atmospheric heavy metal pollution presents a severe threat to public health and environmental stability. Transition metal catalysts have emerged as a potent solution for the selective capture and removal of these pollutants. This review provides a comprehensive summary of current advancements in the field, emphasizing the efficiency and specificity of nanostructured transition metals, including manganese, iron, cobalt, nickel, copper, and zinc. Looking forward, we delve into the prospective trajectory of catalyst development, underscoring the need for materials with enhanced stability, regenerability, and environmental compatibility. We project that advancements in computational materials science, nanotechnology, and green chemistry will be pivotal in discovering innovative catalysts that are economically and environmentally sustainable. The integration of smart technologies for real-time monitoring and adaptive control is anticipated to revolutionize heavy metal remediation, ensuring efficient and responsive pollution abatement strategies in the face of evolving industrial scenarios and regulatory landscapes.

## 1. Introduction

**Figure 1:**
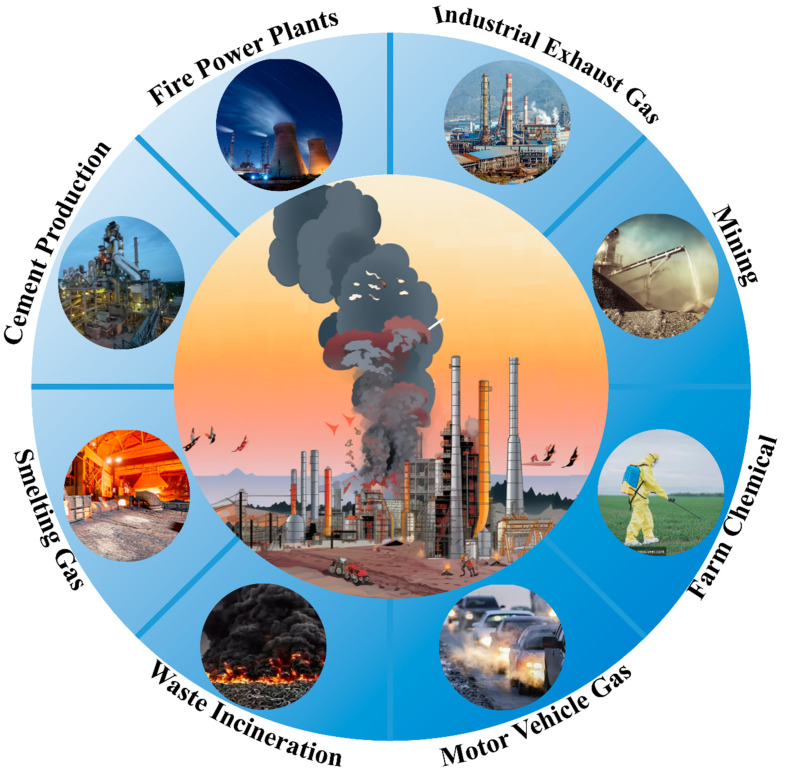
Gaseous heavy metal pollution sources.

Atmospheric heavy metal pollution, a critical public health concern, has intensified with industrialization, emitting toxic elements like arsenic and lead [1]. Industries such as power generation and metal smelting are major emission sources, with coal-fired plants notably contributing to flue gas emissions [2]. High-temperature industrial activities also release heavy metals into the air (Figure 1) [3].

These metals undergo atmospheric transformations, influencing their environmental persistence and human bioaccumulation, with significant health impacts [4]. They contaminate ecosystems and the food chain, necessitating efficient emission capture technologies [5]. Traditional adsorbents lack specificity for certain heavy metals [6], leading to a shift towards nanoscale and porous materials like metal–organic frameworks (MOFs), which offer enhanced selectivity [7].

Heterogeneous catalysis with transition metals has become a key approach for removing atmospheric heavy metals, supported by advancements in catalyst design for improved specificity and cost effectiveness [8]. Research is focusing on bimetallic systems and tailoring catalyst properties for better performance [9]. The aim is to develop scalable, environmentally friendly technologies for industrial applications, translating laboratory innovations into real-world solutions to combat atmospheric heavy metal pollution [10]. The toxicity and health impacts of major atmospheric heavy metal pollutants are given in Table 1.

Transition metals are exceptionally well suited as catalysts for highly selective capture and removal of atmospheric heavy metals. The variable oxidation states, coordination modes and tunable active sites of transition metals like iron, manganese, cobalt, nickel, copper and zinc facilitate specific binding with heavy metal contaminants [11]. The nanoscale particle size and morphology of transition metals can be tailored using synthesis techniques like coprecipitation and sol–gel methods to optimize surface reactivity, porosity and dispersion over supports [12]. Interface engineering with ceria and carbon nanomaterials further augments transition metal nanocatalyst stability and removal efficiency under high temperature and corrosive conditions [13]. Overall, the rich coordination chemistry, structural versatility and multifunctional properties of transition metal catalysts offer tremendous potential for mitigating the escalating crisis of atmospheric heavy metal pollution through rational design. Conventional and emerging technologies for heavy metal removal from atmosphere are given in Table 2.

This review discusses recent advances in emerging transition metal catalytic systems for atmospheric heavy metal removal. The sources, toxicity and environmental impacts of key heavy metal pollutants are first outlined. Progress in the rational design and development of nanostructured transition metal catalysts using synthesis strategies to optimize selective removal performance is then reviewed. Key mechanisms including adsorption, reduction, photocatalytic degradation and vapor-phase catalytic oxidation are examined. Finally, current challenges and future prospects for transition metal catalysts as solutions for real-world atmospheric heavy metal abatement are discussed.

## 2. Transition Metal Nanoparticle Catalysts

**Figure 2:**
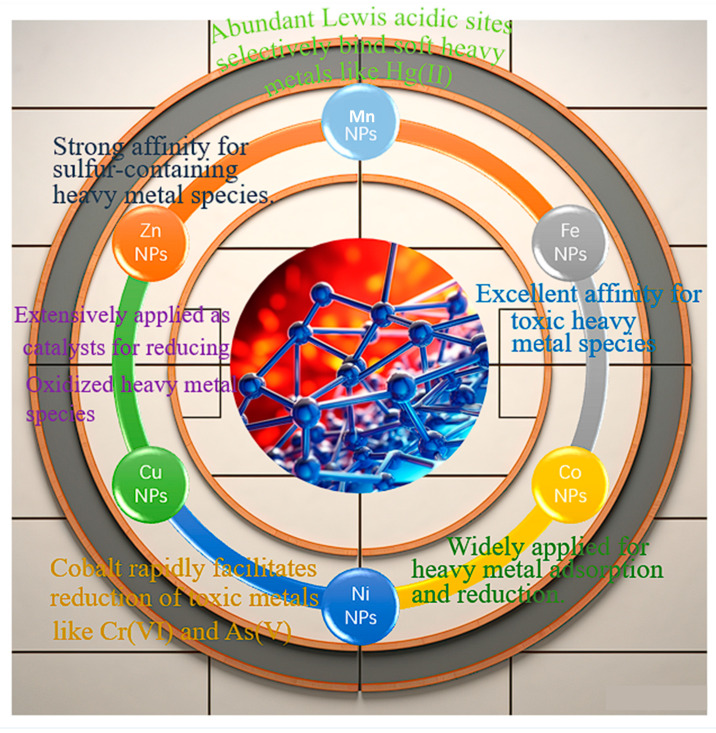
Transition Metal Nanoparticle designs.

Transition metal nanoparticles (NPs) supported over porous materials have been widely explored as promising catalysts for adsorptive removal and reduction in atmospheric heavy metals. Nanostructuring enhances reactive surface area and metal dispersion while supports like silica and activated carbon provide stability and prevent aggregation [14]. By modulating the transition metal identity, nanoparticle size and morphology, and support interactions, catalytic performance for selectively capturing atmospheric heavy metals can be optimized. The most representative NPs are shown in Figure 2 and Table 3.

### 2.1. Supported Manganese Nanoparticles

Manganese nanoparticles (Mn NPs) exhibit excellent affinity for toxic heavy metal species. Islam et al. synthesized mesoporous silica-encapsulated Mn NPs (Mn@mSiO_2_) with sizes of 10–30 nm via a microemulsion technique [15]. The Mn@mSiO_2_ catalyst demonstrated a removal capacity of 193 mg/g for Pb(II) and 322 mg/g for Cd(II) from simulated flue gas. XPS and XAFS analysis revealed that the oxidation of Mn(II) to Mn(III,IV) occurred, indicating redox reactions between the Mn NPs and heavy metals.

### 2.2. Supported Iron Nanoparticles

Iron nanoparticles are widely applied for heavy metal adsorption and reduction due to high capacity, low cost and environmental compatibility [16]. Ultrafine Fe NPs (~3 nm) prepared using a hydrogen reduction approach showed excellent capture of Hg(II) from flue gas [17]. The high oxygen mobility and redox activity of the Fe NPs enabled oxidation of elemental mercury (Hg^0^) to Hg(II) which was rapidly adsorbed. The Fe NPs maintained mercury removal efficiency >90% over 100 h of operation.

### 2.3. Supported Cobalt Nanoparticles

Cobalt possesses variable oxidation states and rapidly facilitates reduction in toxic metals like Cr(VI) and As(V) [18]. Flower-like Co NPs synthesized using diethylene glycol were dispersed on biochar for heavy metal remediation [19]. The Co-biochar composites exhibited a maximum Cd(II) removal capacity of 124 mg/g. XPS and XANES studies determined Cd(II) was partially reduced to Cd(0) by electron transfer from Co(II) sites which was oxidized to Co(III).

### 2.4. Supported Nickel Nanoparticles

Nickel NPs have been extensively applied as catalysts for reducing oxidized heavy metal species due to high reducibility [20]. Ni NPs immobilized on nitrogen-doped graphene showed excellent performance for gas-phase Cr(VI) removal, achieving a 96.6% removal efficiency [21]. In situ DRIFTS and XANES analysis revealed adsorption and reduction of Cr(VI) to Cr(III) occurred on the Ni NP surface.

### 2.5. Supported Copper Nanoparticles

Copper nanoparticles exhibit strong affinity for sulfur-containing heavy metal species. Thiol-functionalized Cu NPs synthesized by NaBH4 reduction were able to capture >99% of SO_2_ along with toxic Hg(II) from simulated flue gas [22]. The Cu NPs showed 10 and 5 times higher Hg(II) adsorption capacity compared to commercial CuO and CuS sorbents, respectively.

### 2.6. Supported Zinc Nanoparticles

Zinc nanoparticles present abundant Lewis acidic sites that selectively bind soft heavy metals like Hg(II) [23]. Porous ZnO supported Zn NPs prepared using a zinc–ethanolamine complex showed excellent capture of elemental mercury from flue gas [24]. The Zn NPs-ZnO catalyst demonstrated a >90% mercury removal efficiency over 180 h of continuous operation. XPS analysis revealed the captured Hg^0^ was oxidized to HgO on the Zn nanoparticle surface.

**Figure 3:**
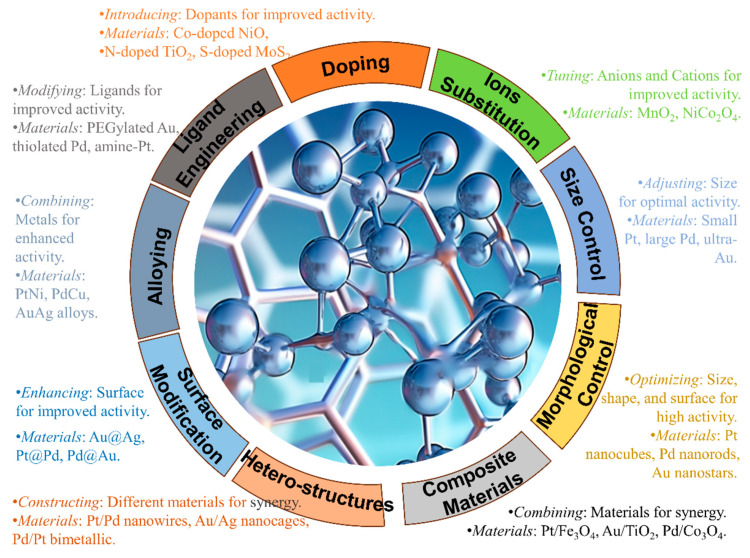
Nanostructured transition metal catalysts design methods.

The overall heavy metal removal performance of transition metal nanocatalysts is dictated by the metal precursor, the preparation method, nanoparticle size and crystallinity, support material and interface interactions [25]. Future work should focus on scalable synthesis techniques to produce sinter-resistant supported transition metal nanoparticles with optimized active facets for selective and efficient capture of toxic atmospheric heavy metals. Nanostructured transition metal catalysts design methods are shown in Figure 3.

**Table 3:** Representative synthesis strategies for nanostructured transition metal catalysts.

Strategy	Description	Materials	Performance Metrics	Reference
Morphological control	Controlling the size, shape, and surface area of nanoparticles to optimize catalytic activity	Pt nanocubes, Pd nanorods, Au nanostars	High catalytic activity, high selectivity	[26]
Anion substitution	Replacing anions in the nanoparticle lattice to tune the electronic structure and improve catalytic activity	MnO_2_, NiCO_2_O_4_	High catalytic activity, high stability	[27]
Cation substitution	Replacing cations in the nanoparticle lattice to tune the electronic structure and improve catalytic activity	CuFe_2_O_4_, CoFe_2_O_4_, MnFe_2_O_4_	High catalytic activity, high stability	[28]
Composite materials	Combining two or more materials to create synergistic effects that enhance catalytic activity and selectivity	Pt/Fe_3_O_4_, Au/TiO_2_, Pd/CO_3_O_4_	High catalytic activity, high selectivity	[29]
Heterostructures	Constructing heterostructures by combining two or more materials with different properties to create synergistic effects that enhance catalytic activity and selectivity	Pt/Pd nanowires, Au/Ag nanocages, Pd/Pt bimetallic nanoparticles	High catalytic activity, high selectivity	[30]
Surface modification	Modifying the surface of nanoparticles to improve catalytic activity and selectivity	Au@Ag core–shell nanoparticles, Pt@Pd core-shell nanoparticles, Pd@Au core-shell nanoparticles	High catalytic activity, high selectivity	[27]
Alloying	Combining two or more metals to create alloys that exhibit enhanced catalytic activity and selectivity	PtNi alloy nanoparticles, PdCu alloy nanoparticles, AuAg alloy nanoparticles	High catalytic activity, high selectivity	[26,30]
Ligand engineering	Modifying the ligands on the surface of nanoparticles to improve catalytic activity and selectivity	PEGylated Au nanoparticles, thiolated Pd nanoparticles, amine-functionalized Pt nanoparticles	High catalytic activity, high selectivity	[26]
Size control	Controlling the size of nanoparticles to optimize catalytic activity and selectivity	Small Pt nanoparticles, large Pd nanoparticles, ultrasmall Au nanoparticles	High catalytic activity, high selectivity	[28]
Doping	Introducing dopants into the nanoparticle lattice to tune the electronic structure and improve catalytic activity	Co-doped NiO, N-doped TiO_2_, S-doped MoS_2_	High catalytic activity, high stability	[30]

## 3. Metal–Organic Frameworks (MOFs)

Metal–organic frameworks (MOFs) have emerged as a versatile class of porous nanomaterials for capturing atmospheric heavy metal pollutants. MOFs are constructed from metal clusters coordinated to organic linkers, creating highly porous structures with ultrahigh surface areas exceeding 7000 m^2^/g [31]. By functionalizing the organic ligands and metal clusters, MOFs can be rationally designed to selectively adsorb specific heavy metal species from complex gas mixtures. MOFs also facilitate photocatalytic degradation of heavy metals under solar irradiation. MOFs synthesis methods categorized by reaction conditions are given in Table 4.

### 3.1. Adsorptive Heavy Metal Removal

MOFs possess abundant unsaturated metal sites and functionalized pores that promote chelation and complexation with heavy metals. Huang et al. synthesized a thiol-functionalized MOF ([Zn2(atb)2(bpee)] (MOF-2)) which showed excellent As(III) and As(V) removal capacity from simulated flue gas [42]. The thiol groups strongly bound arsenic species through As-S coordination. MOF-2 exhibited capacities of 201 mg/g for As(III) and 266 mg/g for As(V), significantly outperforming conventional adsorbents.

### 3.2. Photocatalytic Oxidation

Semiconductor MOFs containing photoactive metal clusters like Ti, Fe, and Zr can generate reactive oxygen species under light irradiation to oxidize toxic heavy metals. MIL-125-NH2, a Ti-based MOF, was able to completely oxidize harmful Hg^0^ vapor under visible light [43]. The photo-induced holes and superoxide radicals efficiently converted Hg^0^ to Hg^2+^, which was captured in the MOF pores, preventing volatilization.

### 3.3. Enhancing Selectivity

MOFs selectivity for target heavy metals can be improved through rational functionalization of the organic linkers. Amino-functionalized MOFs are promising for capturing acidic oxoanions of toxic metals like Cr(VI) and As(V). Li et al. synthesized an ethylenediamine-modulated MOF which showed excellent Cr(VI) removal capacity of 199 mg/g [44]. The amino groups facilitated strong chemisorption and reduction in Cr(VI) to less toxic Cr(III).

In summary, MOFs offer a versatile platform for atmospheric heavy metal capture through highly porous structures, tunable functionality and photocatalytic activity. Further development of visible light responsive, durable MOFs through modular synthesis and advanced characterization will drive progress in atmospheric heavy metal remediation.

## 4. Porous Coordination Polymers (PCPs)

**Figure 4:**
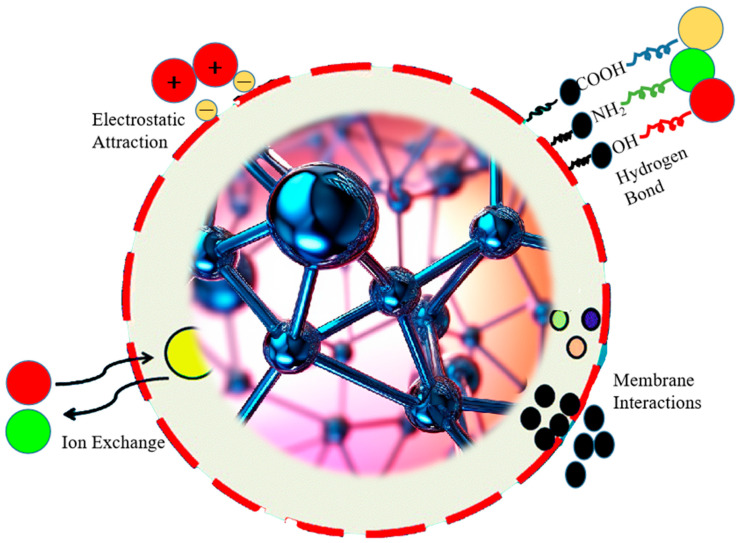
Gaseous heavy metal pollution adsorption.

Porous coordination polymers (PCPs), also known as metal–organic polyhedra (MOPs), have attracted interest as selective sorbents for separating toxic heavy metals like mercury and arsenic from flue gas streams. PCPs consist of multimetallic nodes bridged by organic ligands to create crystalline porous architectures [45]. The tunable pore sizes, high surface areas, and functionalizable pores of PCPs facilitate selective adsorption and separation of heavy metal contaminants [46], the PCPs adsorption of pullutions are shown in Figure 4. Modulating the metal binding sites also imparts redox activity to enable catalytic heavy metal removal.

### 4.1. Pore Size Effects

The ultrafine micropores (<2 nm) and open metal sites in PCPs promote capture of heavy metal ions and particles. Yuan et al. synthesized a series of PCPs with pore sizes ranging from 0.5 to 2.5 nm and demonstrated size selective adsorption of metal ions [47]. The PCP with 0.9 nm pores showed maximum uptake of 387 mg/g for Hg^2+^ compared to lower adsorption of Pb^2+^, Cd^2+^ and Zn^2+^. This high Hg^2+^ selectivity was attributed to complementary interactions between the pore size and the ionic diameter. The porous coordination polymers for heavy metal removal are given in Table 5.

### 4.2. Active Site Modulation

Incorporating coordinatively unsaturated metal sites in PCPs facilitates strong binding with heavy metals. Zhang et al. synthesized an aluminum methylphosphonate PCP containing open Al(III) sites which demonstrated a remarkable arsenic adsorption capacity of 1025 mg/g [53]. XPS and XAFS studies revealed As(III) and As(V) were selectively captured via ligand exchange reactions with surface hydroxyl groups of the Al(III) sites.

### 4.3. Redox Catalysis

The reactive open metal sites in PCPs also enable redox transformations of toxic metals like Hg(II) and Cr(VI). A Ti(IV)-based PCP synthesized by Boorboor et al. exhibited selective oxidation of Hg^0^ and photoreduction of Cr(VI) under UV irradiation [54]. XPS analysis confirmed Hg^0^ was oxidized to HgO while Cr(VI) was reduced to less toxic Cr(III) species by photoexcited electrons. The local coordination environment around the Ti(IV) sites facilitated the photocatalytic activity.

Overall, precise modulation of pores sizes, functional groups, and metal binding sites make PCPs promising selective sorbents for removing toxic heavy metal contaminants from industrial flue gas emissions. Future development of durable PCPs with optimized pore structures and active sites will help advance practical heavy metal remediation applications.

## 5. Metal Oxide Catalysts (MOC)

Metal oxides are a versatile class of catalysts that have shown immense potential for removing toxic heavy metals from industrial flue gas emissions. Redox-active transition metal oxides, especially manganese, iron, cobalt, nickel, copper and zinc oxides, exhibit favorable properties like high stability, good adsorptivity, and multiple oxidation states that facilitate catalytic abatement of atmospheric heavy metals [55].

Nanostructuring enhances the performance of metal oxide catalysts by providing increased reactive surface area, surface defects, and nanoscale interactions that augment heavy metal adsorption and catalytic activity [56]. Common synthetic approaches include sol–gel methods, hydrothermal/solvothermal techniques, precipitation, and gas-phase synthesis which allow tailored design of metal oxide nanocatalyst morphology [13]. Supporting nanostructured metal oxides on porous carriers improves stability and prevents aggregation during high-temperature operation [57]. Interfacing with carbon materials and ceria also modulate oxidation states and oxygen mobility to enhance redox catalysis [58]. The bimetallic catalysts applications for gaseous heavy metal removal are given in Table 6.

### 5.1. Adsorption Mechanisms

Metal oxides demonstrate excellent adsorption capacity for heavy metals due to surface hydroxyl groups, oxygen vacancies, and labile lattice oxygen that can trap metal ions and particles [64]. Nanosizing provides additional unsaturated coordination sites and surface defects that promote heavy metal adsorption through mechanisms like ion exchange and surface complexation [65]. For instance, mesoporous manganese oxide synthesized by Shaheen et al. showed a Pb(II) adsorption capacity of 362 mg/g, attributed to strong surface complex formation [66].

### 5.2. Photocatalytic Oxidation

Semiconducting metal oxides like TiO_2_, ZnO and α-Fe_2_O_3_ catalyze the photooxidation of toxic metals upon band gap excitation under UV or solar irradiation [67]. Photoexcited holes and radicals oxidize elemental metals like Hg^0^ and As(III) into less volatile higher oxidation states that are readily captured [68]. For example, Fe_2_O_3_ nanoparticles prepared using a sol–gel method achieved a 98% photooxidation of Hg^0^ to HgO, enabling flue gas purification [69].

### 5.3. Redox Catalysis

The variable oxidation states of transition metals like Cu, Fe and Co in metal oxides facilitate catalytic reduction in toxic metals like Hg(II), Cr(VI) and As(V) [70]. Redox cycling between lower and higher metal oxide oxidation states drives the reduction. CuO nanowires synthesized hydrothermally could catalytically reduce 96% of Cr(VI) to Cr(III), which was sequestered on the surface [71].

Overall, metal oxide catalysts present versatile, high-performance platforms for removing heavy metals from industrial flue gas emissions through combinations of adsorption, photooxidation and redox catalytic mechanisms. Advances in morphology and interface engineering, visible light harvesting, and multi-functional composite fabrication offer exciting opportunities to leverage these mechanisms for efficient atmospheric heavy metal abatement.

## 6. Bimetallic Catalysts

Bimetallic catalysts comprised of coupled transition metals have emerged as promising materials for removing airborne heavy metal contaminants. The synergistic effects between the constituent metals in bimetallic nanoparticles and porous materials augment their adsorption capacity, redox activity and stability compared to their monometallic counterparts [72]. Strategic selection of metal pairs and tailored synthesis enables optimization of selective removal performance for target heavy metals. The bimetallic catalysts applications for gaseous heavy metal removal are given in Table 7.

### 6.1. Enhanced Adsorption Capacity

Bimetallic catalysts often demonstrate increased heavy metal adsorption capacity owing to the combination of favorable properties from the two metals. Pd-Cu bimetallic nanoparticles supported on multiwalled carbon nanotubes showed excellent capture of elemental mercury, achieving a 96% removal from simulated flue gas [78]. The Pd facilitated oxidative adsorption of Hg^0^ while Cu provided abundant binding sites. The combined effect significantly exceeded their individual performances.

### 6.2. Bifunctional Redox Sites

Incorporating two transition metals with different reduction potentials generates bifunctional sites that can catalyze sequential conversion of heavy metals between multiple oxidation states. Fe-Cu bimetallic nanoparticles synthesized by Liu et al. displayed cooperative redox activity where Fe(III) centers first adsorbed gaseous Hg^0^ which was then oxidized by interfacial Cu(II) sites [79]. This two-step process resulted in potent capture and oxidation of Hg^0^.

### 6.3. Tunable Electronic Properties

The distinct electronic structures of constituent metals in bimetallic catalysts allow modulating band alignment, charge transfer and surface reactivity. Au-Ag bimetallic nanoclusters exhibited plasmon-enhanced photocatalytic oxidation of Hg^0^ under visible light irradiation [80]. The Ag d-band states improved visible light absorption while Au 5d orbitals trapped excited electrons and catalyzed Hg^0^ oxidation.

In summary, bimetallic catalysts present opportunities to strategically couple the unique properties of transition metals to develop multifunctional materials with synergistically enhanced activity, selectivity and lifespan for removing harmful atmospheric heavy metals.

## 7. Zeolites

Zeolites are crystalline aluminosilicate materials comprised of intersecting channels and cavities that make them promising catalysts for capturing airborne heavy metal pollutants. The porous structure, high surface area, and ion exchange capacity of zeolites facilitate adsorption of heavy metal ions and particles [81]. Functionalization with redox-active metals and organic groups can further augment heavy metal removal performance [82]. The material applications of zeolites for heavy metal removal are given in Table 8.

### 7.1. Porous Structure Effects

The microporous channels and cavities in zeolites promote physical adsorption and trapment of heavy metal contaminants. Zeolite Y with a faujasite structure demonstrated high capacity adsorption of 215 mg/g for Cd(II) ions owing to the porous supercages [88]. Enhancing porosity through hierarchical assemblies and mesopores also increases heavy metal accessibility to adsorption sites [89].

### 7.2. Ion Exchange Properties

The aluminosilicate framework of zeolites contains abundant charge balancing cations that can be readily exchanged with heavy metal ions. Zeolite NaY exhibited reversible ion exchange with Pb(II) and Cd(II), enabling cyclic heavy metal removal from simulated flue gas [81]. The cation exchange capacity was optimized by varying the Si/Al ratio.

### 7.3. Redox Functionalization

Incorporating redox-active transition metals like Fe, Ti and Ce introduces reactive sites in zeolites that chemisorb and catalytically oxidize elemental metals like Hg^0^ [90]. Fe-exchanged ZSM-5 zeolite synthesized by Li et al. displayed potent oxidation of Hg^0^ to HgO along with high thermal stability, achieving near complete mercury removal [91].

## 8. Graphene-Based Materials

Graphene and graphene oxide (GO) are emerging support materials for metal and metal oxide nanocatalysts owing to their high surface area, thermal/chemical stability, and mechanical durability [92]. Graphene also facilitates electron transfer to augment redox catalytic activity for heavy metals [93]. Functionalization with organic groups further allows tuning selectivity. The data on graphene-based materials for heavy metal removal are given in Table 9.

### 8.1. Support for Nanocatalysts

Graphene is an ideal support for dispersing metal and metal oxide nanocatalysts while preventing aggregation and leaching during high-temperature operation [100]. Ni nanoparticles immobilized on nitrogen-doped graphene showed excellent Cr(VI) removal capacity of 96.6% from simulated flue gas [101]. Graphene support provided stability under repeated oxidation–regeneration cycles.

### 8.2. Enhanced Electron Transfer

The high conductivity of graphene facilitates electron transfer to supported catalysts, improving redox conversion of absorbed heavy metals. CO_3_O_4_ nanoparticles grown on graphene nanosheets exhibited potent low-temperature catalytic oxidation of Hg^0^ to HgO [102]. Graphene enabled rapid electron transfer at the CO_3_O_4_-graphene interface to boost Hg^0^ oxidation kinetics.

### 8.3. Functionalization

Oxygen functional groups in GO enable selective coordination with heavy metal ions, while restoration of conjugation in reduced GO improves electron mobility [103]. Thiol-functionalized GO synthesized by Huang et al. demonstrated excellent As(III) removal capacity of 227 mg/g through strong As-S binding [104]. The stacked GO structure increased accessibility to the thiol groups.

In summary, graphene-based materials present versatile supports to augment metal/metal oxide nanocatalysts for atmospheric heavy metal removal through carrier, binding site and electron transfer effects.

## 9. Kinetic Modeling Researches

Understanding the kinetics of heavy metal adsorption on MOFs is fundamental to the design of efficient removal processes. Kinetic studies shed light on the rate at which heavy metal ions are immobilized on the MOF surface, impacting overall removal efficiency.

In this section, key aspects of kinetics and modeling applied to MOFs photocatalysis for removing heavy metal ions, organic pollutants and microbes from water are reviewed. The discussion illustrates the multifaceted reaction networks involved and highlights recent efforts to develop integrated kinetics models incorporating coupled adsorption, interfacial transfer, light absorption, contaminant degradation and mass transport effects that influence overall treatment rates and efficiencies.

Langmuir–Hinshelwood model: The Langmuir–Hinshelwood model is a widely used kinetic model that describes the adsorption and reaction kinetics of surface-catalyzed reactions. Recent advancements in Langmuir–Hinshelwood modeling for MOFs have focused on integrating factors such as photonic efficiency, active site availability, intermediates, and competitive adsorption between multiple contaminants. These refinements enhance the model’s accuracy in describing the kinetics of heavy metal adsorption on MOF surfaces [105].

Pseudo-first-order model: The pseudo-first-order kinetic model is a simplified approach used to describe the kinetics of adsorption reactions. It assumes that the rate of adsorption is directly proportional to the concentration of the adsorbate on the surface. Recent studies applying the pseudo-first-order model have explored its applicability to heavy metal adsorption on MOFs. These investigations have provided insights into the initial stages of adsorption and the factors that influence it, such as temperature and initial concentration [106].

Pseudo-second-order model: The pseudo-second-order kinetic model is an alternative approach for modeling adsorption kinetics. It suggests that the rate of adsorption is related to the square of the concentration of the adsorbate on the surface. Recent developments in the pseudo-second-order model have extended its application to heavy metal adsorption on MOFs. This model accounts for the interactions between heavy metal ions and MOF surfaces and provides insights into the kinetics of adsorption processes, including factors like dosage and initial concentration [107].

Elovich model: The Elovich model is a kinetic model commonly used to describe chemisorption processes. It considers the exponential decrease in adsorption rate over time. Recent studies have applied the Elovich model to investigate the kinetics of heavy metal adsorption on MOFs. This model has been refined to account for factors influencing the rate of adsorption, including temperature and the presence of intermediates [108].

Intraparticle diffusion model: The intraparticle diffusion model focuses on the diffusion of adsorbate molecules within the adsorbent particles. It considers the role of particle size in affecting adsorption kinetics. Recent research has incorporated intraparticle diffusion effects into kinetic models for heavy metal adsorption on MOFs. These studies have provided insights into how particle size influences the rate of adsorption and overall removal efficiency [109].

Chick–Watson model: The Chick–Watson model is commonly applied to describe the kinetics of microbial inactivation through photocatalysis. It considers the reduction in microbe concentration over time. Recent advancements in the Chick–Watson model have focused on its application to MOFs-based photocatalytic disinfection. This model has been adapted to describe multi-step damage processes, shedding light on the kinetics of microbe inactivation [110].

Hom model: The Hom model is an empirical approach used to model the kinetics of microbial inactivation during photocatalysis. It relates the reduction in microbe concentration to reaction time. Recent research has applied the Hom model to investigate the kinetics of microbial inactivation using MOFs-based photocatalysis. This model has been adapted to consider factors such as photonic utilization efficiency and transport effects that influence disinfection rates [111]. The kinetic models applied to atmospheric heavy metal removal by transition metal catalysts are given in Table 10.

## 10. Life Cycle Assessment

The application of transition metal catalysts for atmospheric heavy metal removal has made significant strides in recent years. As we evaluate the innovations and advances in this field, it becomes apparent that conducting a comprehensive Life Cycle Assessment (LCA) is indispensable. LCA, a systematic evaluation of the environmental impacts associated with a product, process, or technology throughout its entire life cycle, offers a holistic perspective on the sustainability of transition metal catalysts for heavy metal removal [121].

Advancements in LCA methodologies: One of the pivotal advancements lies in the refinement and diversification of LCA methodologies tailored to this niche. Researchers are increasingly adopting hybrid and consequential LCA approaches, allowing for a more accurate assessment of the cradle-to-grave environmental implications of heavy metal removal processes. These approaches enable the integration of local environmental factors, temporal dynamics, and indirect consequences into the evaluation, resulting in a more comprehensive understanding of the technology’s sustainability [122].

Materials selection and sourcing: LCA has played a crucial role in optimizing the selection and sourcing of materials used in transition metal catalysts. Evaluations encompass aspects such as the extraction of raw materials, the energy-intensive production of catalysts, and the management of waste streams generated during the life cycle. As a result, researchers are better equipped to identify environmentally favorable materials and minimize the ecological footprint of catalyst production [123].

Energy efficiency and emission reduction: Transition metal catalysts are continually evolving to enhance energy efficiency and reduce emissions during heavy metal removal. LCA studies have been instrumental in quantifying the environmental benefits of these innovations. The assessment of energy consumption, greenhouse gas emissions, and other pollutants provides valuable insights into the catalyst’s ecological performance and guides further research into sustainable design [124].

End-of-life considerations: The disposal and recycling of transition metal catalysts have gained prominence within the context of LCA. Understanding the fate of catalysts at the end of their life cycle, including potential reclamation and recycling processes, is vital. LCA has facilitated a more complete evaluation of the environmental implications associated with the disposal of catalysts, thereby promoting strategies for sustainable end-of-life management.

Socioeconomic and health impacts: Beyond environmental considerations, recent LCA studies have increasingly incorporated socioeconomic and health impacts into the assessment framework. Researchers are examining the potential effects of heavy metal removal processes on local communities and workers, as well as the broader societal implications. This holistic approach ensures that the assessment extends beyond environmental boundaries to encompass the broader spectrum of sustainability.

In summary, the integration of Life Cycle Assessment into the evaluation of transition metal catalysts for atmospheric heavy metal removal marks a pivotal advancement in this field. LCA provides a multidimensional view of the technology’s sustainability, encompassing environmental, socioeconomic, and health aspects. As innovation continues to drive this field forward, LCA remains an invaluable tool for guiding research and development efforts towards more sustainable, efficient, and responsible heavy metal removal technologies. The key impact metrics for life cycle assessment of transition metal catalysts are given in Table 11.

## 11. Challenges and Future Outlook

### 11.1. Environmental and Societal Challenges

The deployment of transition metal catalysts for atmospheric heavy metal removal brings forth several complex challenges that necessitate careful consideration as we move forward. These encompass environmental, societal, and technological dimensions, demanding innovative solutions for sustainable progress.

Environmental challenges: The environmental challenges are paramount, as they directly relate to the core goal of mitigating heavy metal pollution. While transition metal catalysts offer substantial promise, their production and deployment must be carefully managed to minimize ecological impacts. Issues such as the environmental footprint of catalyst manufacturing, energy consumption, and greenhouse gas emissions during operation require vigilant attention. Furthermore, the potential release of nanoscale catalyst particles into the environment demands rigorous study to ensure long-term ecological safety.

Societal and health concerns: Transition metal catalysts for heavy metal removal often operate in urban and industrial settings. Consequently, the health and safety of nearby communities and workers emerge as vital considerations. Ensuring that these technologies do not inadvertently introduce health risks or disproportionately affect vulnerable populations is of paramount importance. Assessing potential socioeconomic disparities and addressing them proactively is a challenge that the field must confront.

### 11.2. Technological Challenges

Scaling up and integration: Transitioning from laboratory-scale experimentation to real-world application remains a significant challenge. Scaling up catalyst production, maintaining performance consistency, and integrating these technologies into existing industrial processes require innovative engineering solutions. Addressing issues like catalyst stability under varying conditions, efficient mass transport, and reactor design are paramount to success.

Long-term stability: The durability and stability of transition metal catalysts in harsh environmental conditions are essential for sustained heavy metal removal. Overcoming issues related to catalyst degradation and ensuring long-term functionality pose notable technological hurdles. Strategies involving materials engineering and advanced coatings are being explored to enhance catalyst longevity.

Resource availability: Many transition metal catalysts rely on critical or scarce elements for their functionality. Ensuring a stable supply of these materials is crucial. The development of alternative catalysts with reduced reliance on rare resources or the implementation of effective recycling and reclamation strategies is essential for long-term viability.

### 11.3. Future Outlook

Outlook for Transition Metal Catalysts:

The future of transition metal catalysts in the context of atmospheric heavy metal removal is poised at the cusp of transformative advancements. As we navigate towards more sustainable and efficient environmental remediation technologies, several key development trajectories are anticipated to shape the evolution of these catalysts.

Material Innovation and Synthesis:

Future research is expected to focus on the discovery and synthesis of novel materials with enhanced catalytic properties. The use of high-throughput computational screening and machine learning will accelerate the identification of catalysts with superior adsorption capacities, selectivity, and stability. Green synthesis routes, employing biological or recycled feedstocks, are projected to gain traction, minimizing the environmental impact associated with catalyst production.

Catalyst Architecture and Nano-engineering:

Advancements in nanotechnology will enable the precise engineering of catalysts at the atomic level, controlling their morphology, surface properties, and porosity. Such nano-engineered catalysts will exhibit higher efficiencies and targeted action, ensuring the rapid and selective removal of heavy metals from complex atmospheric matrices.

Stability and Regeneration:

Ensuring the long-term stability of catalysts under operational conditions remains a critical objective. Research will likely advance towards the development of self-healing and regenerative catalyst systems that maintain their activity over extended periods, reducing the need for replacement and minimizing waste generation.

Integration with Sensing and Monitoring Technologies:

The convergence of catalysis with sensor technology will lead to the advent of intelligent remediation systems. These systems will dynamically adjust to fluctuating pollution levels, optimizing heavy metal capture in real time and providing continuous monitoring to ensure compliance with environmental regulations.

Scalability and Process Integration:

Translating benchtop innovations to industrial-scale applications will be a significant focus. Addressing the challenges of catalyst scalability, including manufacturing and deployment costs, will be key to their widespread adoption. Modular and adaptable catalyst designs will facilitate seamless integration into existing industrial infrastructures.

Holistic Environmental Impact Assessments:

Life Cycle Assessments (LCAs) will become integral in evaluating the environmental footprint of catalysts throughout their lifecycle. Emphasis on the sustainable procurement of raw materials, energy efficient production processes, and end-of-life recycling or disposal will guide the development of environmentally benign catalyst systems.

Policy-Driven Catalyst Development:

Evolving environmental policies and regulations will continue to drive the innovation of transition metal catalysts. Catalyst development will increasingly align with regulatory frameworks aimed at mitigating heavy metal pollution, fostering the creation of compliant and future-proof technologies.

In the foreseeable future, transition metal catalysts will likely emerge as more than mere agents for pollution mitigation; they will become integral components of a broader ecosystem aimed at fostering sustainable industrial practices and promoting public health and environmental conservation. The progressive integration of these catalysts into global efforts to combat atmospheric pollution will exemplify the harmonious intersection of science, technology, and policy, crafting a cleaner and safer environment.

## 12. Conclusions

In conclusion, our review synthesizes the significant strides made in employing transition metal catalysts for the remediation of atmospheric heavy metals, delineating their operational mechanisms, efficiency, and current technological standing. As we peer into the future, we envision a landscape where the development of transition metal catalysts is propelled by breakthroughs in material science, with a pronounced emphasis on sustainability and environmental stewardship. The advent of smart remediation systems, powered by the confluence of catalytic technology and IoT, is set to provide adaptive and precise pollution control. Furthermore, the adoption of green synthesis approaches and life cycle assessments will ensure that catalyst production aligns with global sustainability targets. The challenges of scalability and integration with existing infrastructures will necessitate a collaborative effort across scientific disciplines and industries. By embracing these future directions, transition metal catalysts will not only continue to mitigate the perils of heavy metal pollution, but also pave the way for a cleaner, safer, and more sustainable future.

## Figures and Tables

**Table 1:** Toxicity and health impacts of major atmospheric heavy metal pollutants.

Heavy Metal	Sources	Chemical Forms	Toxicity Mechanisms	Exposure Risks	Associated Health Effects
Lead (Pb)	Mining, smelting, battery manufacturing, paint pigments	Inorganic (Pb^2+^), organic (tetraethyl lead)	Interference with heme synthesis and neurotransmitter release; oxidative stress; DNA damage	Inhalation of lead dust or fumes; ingestion of contaminated food or water; dermal contact with lead-containing substances	Neurological damage; developmental delays; anemia; hypertension
Mercury (Hg)	Coal-fired power plants, mining, dental amalgams	Elemental (Hg^0^), inorganic (Hg^2+^), organic (methylmercury)	Inhibition of enzymes involved in heme synthesis and antioxidant defense; oxidative stress; DNA damage	Inhalation of mercury vapor or dust; ingestion of contaminated fish or water; dermal contact with mercury-containing substances	Neurological damage; developmental delays; kidney damage
Cadmium (Cd)	Mining, smelting, battery manufacturing	Inorganic (Cd^2+^), organic (cadmium chloride)	Interference with calcium signaling and antioxidant defense; oxidative stress; DNA damage	Inhalation of cadmium fumes or dust; ingestion of contaminated food or water; dermal contact with cadmium-containing substances	Kidney damage; osteoporosis
Chromium (Cr)	Electroplating, leather tanning, stainless steel production	Inorganic (Cr^3+^, Cr^6+^), organic (chromium picolinate)	Interference with DNA repair and antioxidant defense; oxidative stress; DNA damage	Inhalation of chromium fumes or dust; ingestion of contaminated food or water; dermal contact with chromium-containing substances	Lung cancer
Arsenic (As)	Mining, smelting, pesticide production	Inorganic (As^3+^, As^5+^), organic (arsenobetaine)	Interference with ATP production and antioxidant defense; oxidative stress; DNA damage	Inhalation of arsenic dust or fumes; ingestion of contaminated food or water; dermal contact with arsenic-containing substances	Skin cancer
Thallium (Tl)	Coal-fired power plants, cement production, electronics manufacturing	Inorganic (Tl^+^)	Interference with potassium channels and antioxidant defense; oxidative stress; DNA damage	Inhalation of thallium dust or fumes; ingestion of contaminated food or water; dermal contact with thallium-containing substances	Nerve damage

**Table 2:** Conventional and emerging technologies for heavy metal removal from atmosphere.

Method	Materials	Advantages	Disadvantages	Future Directions
Adsorption	Activated carbon, zeolites, chitosan, etc.	High efficiency, low cost, easy operation	Limited reusability, low selectivity	Development of novel adsorbents
Photocatalysis	TiO_2_, ZnO, Fe_2_O_3_, etc.	High efficiency, no secondary pollution	Limited light absorption range, high cost	Development of visible-light-responsive photocatalysts
Membrane separation	Ultrafiltration membranes, nanofiltration membranes, reverse osmosis membranes, etc.	High selectivity and efficiency	Membrane fouling, high energy consumption	Development of antifouling membranes
Ion exchange	Resins, zeolites, etc.	High selectivity and efficiency	Limited reusability, low capacity for heavy metals with low concentrations	Development of novel ion exchangers
Electrochemical treatment	Electrodes (Fe, Al), electrolytes (NaCl), etc.	High efficiency and selectivity, no secondary pollution	High energy consumption, limited application range	Development of novel electrode materials
Phytoremediation	Plants (e.g., sunflower, Indian mustard)	Low cost, eco-friendly, aesthetically pleasing	Limited application range, slow process	Development of plants with higher heavy metal uptake capacity
Bioremediation	Microorganisms (e.g., bacteria, fungi)	Low cost, eco-friendly, high efficiency	Limited application range, slow process	Development of microorganisms with higher heavy metal uptake capacity
Coagulation/flocculation	Coagulants (e.g., alum, ferric chloride), flocculants (e.g., polyacrylamide)	High efficiency, easy operation	High chemical consumption, secondary pollution	Development of novel coagulants/flocculants
Electrocoagulation	Electrodes (e.g., iron, aluminum), electrolytes (e.g., NaCl)	High efficiency and selectivity, no secondary pollution	High energy consumption, limited application range	Development of novel electrode materials

**Table 4:** MOFs synthesis methods categorized by reaction conditions.

MOFs	Method	Mechanism	Capacity	Target	Reuse Cycles	References
UiO-66-NH_2_	Solvothermal	Photoreduction	198.7 mg/g	Pb(II)	4	[32]
Cu-BTC	Hydrothermal	Photoreduction	167.2 mg/g	Hg(II)	5	[33]
Cd-MOFs	Microwave assisted	Photoreduction	71.4 mg/g	Cr(VI)	3	[34]
MIL-53	Ultrasonication	Photooxidation	92.6 mg/g	Methyl orange dye	6	[35]
Zn-MOFs	Solvothermal	Photooxidation	248.7 mg/g	Rhodamine B dye	4	[36]
NH_2_-MIL-125	Solvothermal	Photoreduction	175.4 mg/g	Ag(I)	5	[37,38]
ZIF-8	STP synthesis	Photooxidation	104.7 mg/g	Methylene blue dye	3	[38]
Cu-BTC/GO	Hydrothermal	Photoreduction	152.6 mg/g	Cd(II)	4	[39]
Fe-MIL-101	Solvothermal	Photoreduction	198.4 mg/g	Cr(VI)	3	[40]
UiO-66	Microwave assisted	Photooxidation	167.9 mg/g	Orange II dye	5	[41]

**Table 5:** Porous coordination polymers for heavy metal removal.

PCP Composition	Pore Size	Target Heavy Metal	Removal Capacity	Mechanism	Ref.
Zn-based PCP	0.9 nm	Hg^2+^	387 mg/g	Size-selective adsorption	[48]
Al-methylphosphonate PCP	Microporous	As(III), As(V)	1025 mg/g	Ligand exchange at Al(III) sites	[49]
Ti(IV)-based PCP	<2 nm	Hg^0^	-	Photooxidation to HgO	[50]
Ti(IV)-based PCP	Microporous	Cr(VI)	-	Photoreduction to Cr(III)	[51]
Fe-azolate PCP	1.5 nm	Hg^2+^	296 mg/g	Coordinative binding at metal sites	[52]

**Table 6:** Bimetallic catalysts applications for gaseous heavy metal removal.

Bimetallic Catalyst	Properties	Application	Reference
TiO_2_	Bandgap: ~3.2 eV—High photocatalytic activity—Stability under UV light	Removal of Cr(VI), Cd(II), and organic pollutants—Water and wastewater treatment	[59]
ZnO	Bandgap: ~3.37 eV—Good photocatalytic efficiency—Low cost	Degradation of organic dyes and heavy metals—Environmental remediation	[60]
Fe_2_O_3_	Hematite structure—High surface area—Photocorrosion resistance	Elimination of arsenic, chromium, and lead—Groundwater purification	[61]
CuO	Cupric oxide—Visiblelight absorption—Antibacterial properties	Copper ion removal—Microbial disinfection—Industrial wastewater treatment	[62]
SnO_2_	Tin dioxide—Stable and nontoxic—Photocatalytic activity—Wide bandgap	Removal of Hg(II), As(III), and Cr(VI)—Air and water purification	[63]

**Table 7:** Bimetallic Catalysts Applications for Gaseous Heavy Metal Removal.

Bimetallic Catalyst	Composition	Properties	Application	Reference
AuAg Nanoparticles	AuAg alloy	Synergistic effect—Enhanced catalytic activity—High stability	Removal of Hg(II) and Cr(VI)—Water treatment	[73]
PdCu Nanoparticles	PdCu alloy	High catalytic selectivity—Improved electron transfer—Resistance to deactivation	Degradation of chlorinated organics—Groundwater remediation	[74]
PtFe Nanoparticles	PtFe alloy	Excellent catalytic performance—Magnetic properties—Good reusability	Reduction of nitroaromatic compounds—Soil and sediment remediation	[75]
NiCo Nanoparticles	NiCo alloy	Tunable composition—High surface area—Enhanced adsorption	Removal of heavy metals from industrial effluents—Environmental cleanup	[76]
CuAg Nanoparticles	CuAg alloy	Surface plasmon resonance—Photocatalytic activity—Low toxicity	Disinfection of microbes—fluegas and wastewater treatment	[77]

**Table 8:** Zeolite material applications for Heavy Metal Removal.

Zeolite Type	Composition	Properties	Application	Ref.
Natural Clinoptilolite	Aluminosilicate	High cation exchange capacity Selective adsorption Low cost	Removal of ammonium and heavy metals, Soil and water remediation	[83]
NaX Zeolite	Sodium aluminosilicate	Large surface area High ionexchange capacity Selective adsorption	Removal of Cs, Sr, and heavy metals from nuclear wastewater Water purification	[84]
Ferrierite Zeolite	Aluminosilicate	Acidic properties Mesoporous structure High thermal stability	Adsorption of heavy metals and organic pollutants Catalytic applications	[85]
Mordenite Zeolite	Aluminosilicate	Long pore channels High aluminum content Excellent stability	Removal of ammonium and heavy metals from wastewater Catalytic processes	[86]
Faujasite Zeolite	Aluminosilicate	Cage-like structure Acidic sites Hydrophobic and hydrophilic regions	Adsorption of heavy metals and organic compounds Petroleum refining	[87]

**Table 9:** Graphene-Based Materials for Heavy Metal Removal.

Material Type	Composition	Properties	Application	Reference
Graphene Oxide (GO)	Carbon-based	Large surface area	Adsorption of heavy metals and organic pollutants Water and wastewater treatment	[94]
Reduced Graphene Oxide (rGO)	Reduced form of GO	Restored conductivity Enhanced adsorption properties	Removal of heavy metals from industrial effluents Electrochemical sensors	[95]
Graphene-Based Nanocomposites	Graphene combined with other materials (e.g., nanoparticles)	Synergistic properties Improved stability	Efficient removal of heavy metals Environmental remediation	[96]
Graphene Nanosheets	Two-dimensional carbon structure	High surface area Enhanced reactivity	Adsorption and catalytic degradation of pollutants Water purification	[97,98]
Graphene Quantum Dots	Quantum-sized graphene particles	Size-dependent properties Excellent photoluminescence	Sensing and detection of heavy metals Bioimaging applications	[99]

**Table 10:** Kinetic models applied to atmospheric heavy metal removal by transition metal catalysts.

Materials	Metals	Kinetic Model	Rate Expression	Reference
-TiO_2_ nanocomposites	Hg^0^	Langmuir–Hinshelwood	r = kθC/(1 + KC)	[112]
Fe-ZSM-5 zeolite	Hg^0^	Pseudo-first-order	ln(C_0_/C) = k’t	[113]
Ni-CeO_2_ nanocatalyst	Pb(II)	Pseudo-second-order	t/Qt = 1/k’Q_e_^2^ + t/Q_e_	[114]
MOF-5	Cd(II)	Elovich	qt = (1/β)ln(αβ) + (1/β)lnt	[115,116]
Co@Co_3_O_4_ core-shell	As(III)	Intraparticle diffusion	qt = k’√t	[117,118]
Ag-ZnO nanoparticles	Hg^0^	Chick–Watson	ln(N/N_0_) = −k’C’t	[115]
Pd-Cu bimetallic catalyst	Hg^0^	Hom model	ln(N/N_0_) = −k’C’ntm^−1^	[119,120]

**Table 11:** Key impact metrics for life cycle assessment of Transition Metal Catalysts.

Impact Metric	Description
Embodied energy	Energy utilized for materials synthesis and processing
GWP	Greenhouse gas emissions across life cycle
Eutrophication potential	Impacts on aquatic ecosystems from discharges
Human health criteria	Exposure to hazardous substances
Material use	Consumption of resources, recyclability
Synthesis greenness	Use of biogenic/waste precursors, benign solvents
Stability/reusability	Lifetime, metal leaching, structural integrity

## Nomenclature

C	Concentration of contaminant; mg × L^−1^
C_0_	Initial concentration of contaminant; mg × L^−1^
k	Reaction rate constant; varies
kr	Intrinsic reaction rate constant; s^−1^
K	Adsorption equilibrium constant; L × mg^−1^
t	Irradiation time; min
r	Reaction rate; mg × L^−1^∙min
θ	Surface coverage; dimensionless
q	Adsorbed amount; mg × g^−1^
Q_t_	Adsorbed amount at time t; mg × g^−1^
Q_e_	Adsorbed amount at equilibrium; mg × g^−1^
N	Number of viable microbes at time t
N_0_:	Initial number of viable microbes
F	Photon flux; mW × cm^−2^
D	Diffusion coefficient; m^2^ × s^−1^
dp	Particle diameter; m
